# High Quality Plasmonic Sensors Based on Fano Resonances Created through Cascading Double Asymmetric Cavities

**DOI:** 10.3390/s16101730

**Published:** 2016-10-18

**Authors:** Xiangao Zhang, Mingzhen Shao, Xiaoqi Zeng

**Affiliations:** Department of Physics, South University of Science and Technology of China, Shenzhen 518055, China; shaomz@sustc.edu.cn (M.S.); zengxq@sustc.edu.cn (X.Z.)

**Keywords:** reflective index sensor, plasmonics, Fano resonance, metal-insulator-metal waveguide

## Abstract

In this paper, a type of compact nanosensor based on a metal-insulator-metal structure is proposed and investigated through cascading double asymmetric cavities, in which their metal cores shift along different axis directions. The cascaded asymmetric structure exhibits high transmission and sharp Fano resonance peaks via strengthening the mutual coupling of the cavities. The research results show that with the increase of the symmetry breaking in the structure, the number of Fano resonances increase accordingly. Furthermore, by modulating the geometrical parameters appropriately, Fano resonances with high sensitivities to the changes in refractive index can be realized. A maximum figure of merit (FoM) value of 74.3 is obtained. Considerable applications for this work can be found in bio/chemical sensors with excellent performance and other nanophotonic integrated circuit devices such as optical filters, switches and modulators.

## 1. Introduction

Plasmonics have attracted considerable research interest because of their special capability to confine light to subwavelength dimensions and overcome the traditional optical diffraction limit [1,2,3,4,5,6,7]. As we know, there has been remarkable progress in developing plasmonic nanostructures, such as surface-enhanced Raman spectroscopy [8], bio/chemical sensors [9,10,11,12], optical waveguides [13,14], lasers [15], absorbers [16], reflectors [17,18], and logic devices [19,20]. Recently, much attention has been given to plasmonic Fano resonances [21,22,23,24], which are classical analogues of the Fano resonance of a quantum system interfered with the discrete excited state of an atom with a continuous state. Different from Lorentz resonances (symmetric spectra), Fano resonances generally exhibit asymmetric lineshapes.

Fano resonances sustained by plasmonic structures depend strongly on the shape and size of the geometry and surrounding materials. Such properties have potential applications in designing plasmonic devices, especially for various nanosensors. It has been demonstrated that this type of sensor can be designed in a metal-insulator-metal (MIM) waveguide and an asymmetric rectangular cavity, in which independently tunable double Fano resonances can be achieved by changing the different parameters of the geometry [25]. Moreover, it has been reported that Fano-type sensors with high sensitivities can be realized in nanoshell clusters deposited on a substrate of *β*-SiC/SiO_2_/Si multilayers, indicating that the multilayer substrate plays a fundamental role in the confinement of optical power in the nanoshell layer and results in the formation of pronounced Fano dips [26]. Furthermore, refractive index sensors with high-order Fano resonances have also been demonstrated in dielectric-core-metal-shell and metal-core-dielectric-shell configurations. The results show that the designs can provide a high modulation depth for multi frequency sensors [27]. From above, we can see that Fano resonances play an important role in designing and modulating plasmonic sensors.

As we know, optimizing the plasmonic structures and enhancing the transmission efficiency is a challenge when designing nanosensors. In order to realize high-quality sensors with the two above-mentioned features, we propose a new type of compact plasmonic sensor realized by cascading double asymmetric cavities at the center of a MIM waveguide. The purpose of using cascaded structures is to obtain high-sensitivity sensing through enhancing the mutual coupling of two cavities. By modulating the geometrical parameters appropriately, multiple Fano resonances with high transmission and sharp lineshapes can be obtained by breaking the symmetry in the cascaded cavities. Such properties considered here can be found in many important applications in various bio/chemical sensors, and other nanophotonic integrated circuit devices such as optical filters, switches, and modulators.

## 2. Structure Descriptions and Theory Analysis

**Figure 1:**
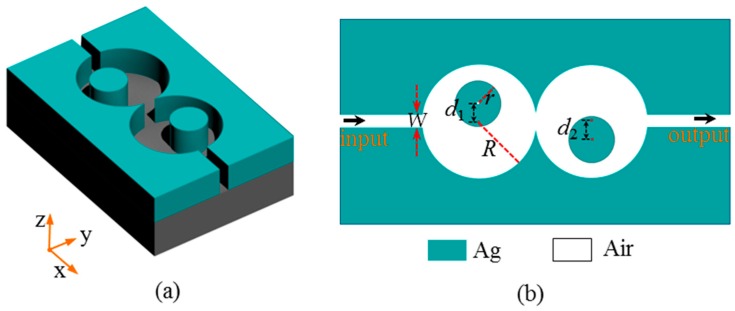
(**a**) A metal-insulator-metal (MIM) nanostructure composed of a main waveguide and two cascaded asymmetric cavities at the center; (**b**) the corresponding cross section of the structure where the left and right metal cores of the cavities shift along +*y* and −*y*-axis directions with distances of *d*_1_ and *d*_2_, respectively.

In general, there are two types of plasmonic structures used for designing devices: the insulator-metal-insulator (IMI) and MIM structures. As we know, due to the strong capability to confine light, the MIM structure has more applications in optical devices [28]. Here, we use the MIM structure to design a plasmonic sensor as shown in Figure 1a. It is a two-dimensional nanostructure composed of a main waveguide and two cascaded asymmetric cavities at the center. The corresponding cross section is shown in Figure 1b. Note that, the line shape is usually wide for a single-cavity structure [29]. In order to obtain sharp-peak resonance, we propose a cavities-cascaded structure to strengthen the resonances and interactions of the system. In addition, Fano resonance can always be realized by breaking symmetry in structure. Therefore, in the cavities, the positions of the metal cores are moved along different axis directions to study the Fano properties.

The width of the waveguide is set as *W* = 50 nm. The two cavities have the same radius, denoted by *R*. We chose *R* = 200 nm. Each cavity includes a center-deviated metal core with a radius of *r* = 90 nm. The left and right metal cores shift along the +*y* and −*y*-axis directions with deviation distances of *d*_1_ and *d*_2_, respectively. Here, *d*_1_ and *d*_2_ have the same value *d*, i.e., *d = d*_1_ *= d*_2_. Therefore, the parameter *d* can be used to describe the symmetry breaking in the structure. The insulators in the cavities and waveguide are chosen to be air (*n* = 1.0). The metal is silver, whose frequency-dependent complex relative permittivity is characterized by the Drude model [30,31]:

(1) $$\varepsilon_{m}(\omega )=\varepsilon_{\infty }-\frac{\omega_{p}^{2}}{\omega (\omega +i\gamma )}$$

where *ε_∞_* is the dielectric constant at the infinite frequency, *γ* is the electron collision frequency, *ω* is the frequency of the incident light and *ω_p_* is the bulk plasma frequency. The parameters are *ε_∞_* = 3.7, *ω_p_* = 1.38 × 10^16^ Hz and *γ =* 2.73 × 10^13^ Hz. In order to excite the surface plasmon polaritons, the input light is set to be a transverse magnetic plane wave.

**Figure 2:**
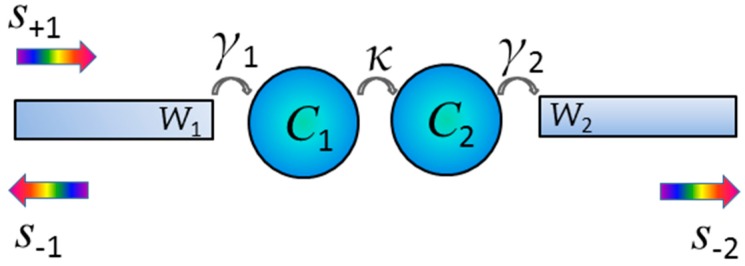
Simplified model of two cascaded cavities coupled with a main waveguide.

In order to understand the theory of the cascaded structure, the temporal coupled mode theory [32,33,34] is utilized to analyze the Fano resonance. As shown in Figure 2, the two cascaded cavities and their connecting waveguide arms are denoted by C*_i_* and W*_i_* (*i* = 1, 2), respectively. For the harmonic time dependence of *e^−jωt^*, where *j* is the imaginary number, the time evolution amplitudes in cavity C*_i_* can be described by *a_i_* (*i* = 1, 2). We have:

(2) $$\frac{da_{1}}{dt}=\left(j\omega_{1}-\gamma_{1}\right)a_{1}+j\sqrt{2\gamma_{1}}s_{+1}-j\kappa a_{2}$$

(3) $$\frac{da_{2}}{dt}=\left(j\omega_{2}-\gamma_{2}\right)a_{2}+j\sqrt{2\gamma_{2}}s_{+2}-j\kappa a_{1}$$

(4) $$s_{-i}=-s_{+i}+j\sqrt{2\gamma_{i}}a_{i}\;\begin{array}{c} \left(i=1,2\right) \end{array}$$

where *s*_±*i*_ (*i* = 1, 2) denote the input and output from the waveguide arm *W_i_*, *ω_i_* is the resonator frequency of the cavity *C_i_*, *γ_i_* is the coupling coefficient between the cavity *C_i_* and its connecting waveguide arm *W_i_*, and *κ* is the mutual coupling coefficient between the two cavities. The transmission *T* from the left to right port can be calculated from Equations (2)–(4) as:

(5) $$T={\left|\frac{s_{-2}}{s_{+1}}\right|}^{2}={\left|\frac{2j\kappa \sqrt{\gamma_{1}\gamma_{2}}}{\left(j\omega -j\omega_{1}+\gamma_{1}\right)\left(j\omega -j\omega_{2}+\gamma_{2}\right)+\kappa^{2}}\right|}^{2}$$

Equation (5) indicates that the transmission from the left to right port is significantly affected by the coupling between the two cascaded cavities *C*_1_ and *C*_2_ (see Figure 2), and between the cavity *C*_1_ (*C*_2_) and the waveguide arm *W*_1_ (*W*_2_). When the asymmetry variable *d* is increased, the coupling between the cavities and waveguide arms will produce the corresponding changes, i.e., the parameters *γ*_1_, *γ*_2_ and *κ* change. It will bring a complex variation (i.e., producing more resonance peaks) for the transmission spectra. Fano phenomena can be formed by the complex resonances and interactions in the coupling system. The introduction of asymmetry to the structure can effectively modulate the cavity coupling, which results in corresponding changes in the Fano resonances. Next, the COMSOL software (finite element method) is used to numerically investigate the transmission properties of the designed structures. In addition, perfectly matched layers are added outside of the calculated domain to absorb the electromagnetic wave. The fundamental TM mode of the plasmonic waveguide is excited at the input port. Two power monitors are set at the input and output port to detect the incident power *P*_in_ and the transmitted power *P*_out_. The transmission is calculated as *P*_out_/*P*_in_ for each wavelength.

## 3. Results and Discussion

**Figure 3:**
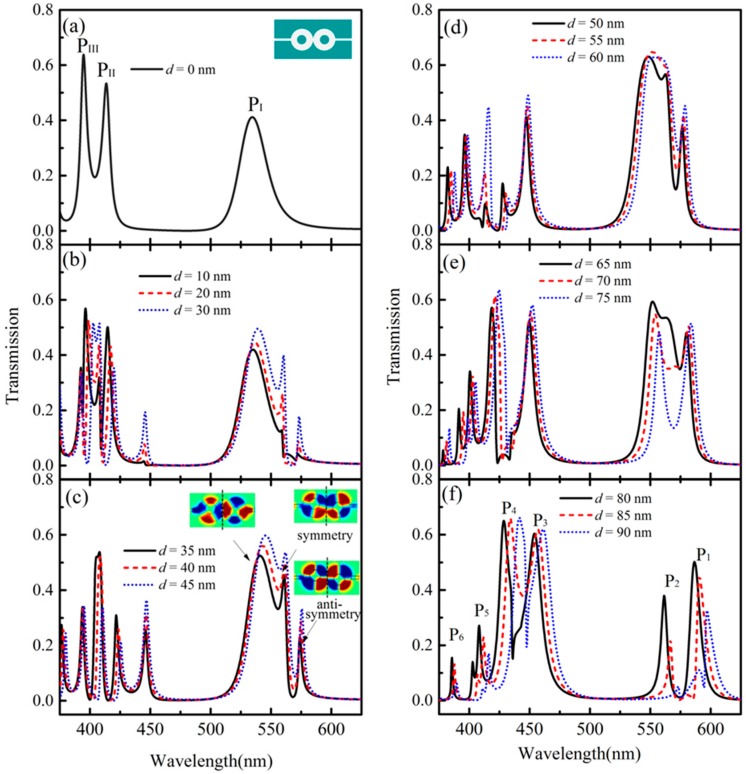
Transmissions for the cases of (**a**) the symmetric structure with the left and right metal cores located at the center of the corresponding cavity, i.e., *d* = 0; and the asymmetric structure with the left and the right metal cores respectively deviating along +*y* and −*y*-axis direction, but with the same deviation distance of *d*; (**b**) *d* = 10, 20, and 30 nm; (**c**) *d* = 35, 40, and 45 nm; (**d**) *d* = 50, 55, and 60 nm; (**e**) *d* = 65, 70, and 75 nm; and (**f**) *d* = 80, 85, and 90 nm.

In order to investigate the impact of structure asymmetry (*d* ≠ 0) on the transmission properties, for a comparison, the case of a symmetric structure (*d* = 0) is first considered, whose transmission is calculated in Figure 3a. The corresponding structure is shown in the inset. From the figure we can see that there exist three transmission peaks in the spectrum, denoted by *P*_I_, *P*_II_, and *P*_III_. It can be considered that they are formed by the resonances and interactions between the two cascaded cavities.

In order to clearly show the transmission changes for different values of *d*, the calculated spectra are divided into three groups according to the ranges for *d*. The transmissions for the cases of Group 1 (*d* = 10, 20 and 30 nm), Group 2 (*d* = 35, 40 and 45 nm), Group 3 (*d* = 50, 55 and 60 nm), Group 4 (*d* = 65, 70 and 75 nm), and Group 5 (*d* = 80, 85 and 90 nm) are given in Figure 3b–f, respectively. Notice in Figure 3 that we can find that multiple Fano resonance peaks with asymmetric lineshapes are formed with the increase of *d* from 0 to 90 nm. When the value of *d* is small (see Figure 3b,c), some new resonance peaks are formed, such as the peak *P*_I_ split into three new peaks. We choose the case of *d* = 35 nm as an example. The modes of the three peaks split from *P*_I_ are shown in the insets of Figure 3c. Specially, for the peaks at *λ*_1_ = 560.7 nm and *λ*_2_ = 574.2 nm, their wavelengths are so close that a deep gap is in the spectrum. In addition, we can find that their modes exhibit symmetry and anti-symmetry related to the central axis (the dotted line), as shown in the insets of Figure 3c. Directly comparing the two figures of Figure 3a,f, it is evident that the resonance peak *P*_I_ produces two new resonance peaks, denoted by *P*_1_ and *P*_2_, and the resonance peaks *P*_II_ and *P*_III_ together produce four new resonance peaks, denoted by *P*_3_, *P*_4_, *P*_5_, and *P*_6_. On the other hand, we can also find that the new resonance peaks *P*_1_ and *P*_2_ have a higher transmission and sharper lineshape than those of the resonance peak *P*_I_. Meanwhile, the new resonance peaks *P*_3_ and *P*_4_ have higher transmissions and sharper lineshapes than those of the resonance peaks *P*_II_ and *P*_III_. It is considered that the deviation of the metal cores (i.e., *d* ≠ 0) will bring more space for the lower part in cavity *C*_1_ and the upper part in cavity *C*_2_. With the asymmetry variable *d* increase, the coupling between the cavities and waveguide arms will produce the corresponding changes. As a result, the parameters of *γ*_1_, *γ*_2_ and *κ* in Equation (5) will change. It will bring a complex variation for the transmission spectra. To a certain extent, it can increase the connectivity between the two cascaded cavities, which can further strengthen the resonances and interactions of the system coupling. Thus, some new Fano resonance modes are introduced because of the complex coupling, and the transmissions can be enhanced.

**Figure 4:**
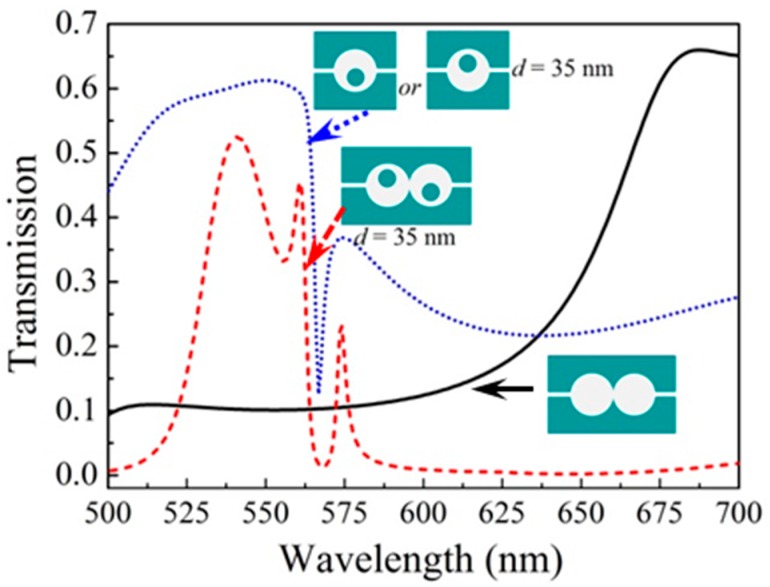
Transmissions for three kinds of different structures.

In order to investigate the mechanism of the Fano resonance, two types of other structures are considered. One is double cavities without metal cores and the other is single cavity with a center-deviated metal core (*d* = 35 nm). The corresponding transmissions are shown in Figure 4. From the figure, we can see that there is a wide spectrum for double cavities without metal cores (solid line), which can be regarded as “the continuum of state”. Moreover, there is a sharp-peak spectrum for single cavity with a center-deviated metal core (dotted line), which can be regarded as “the discrete state”. For comparison, the transmission of asymmetric double-cavities structure (i.e., the spectrum for the case of *d* = 35 nm shown in Figure 3c) is also given in Figure 4 (dashed line). We can consider that the Fano resonance arises from the coupling interaction of the continuum state and the discrete state [35,36].

**Figure 5:**
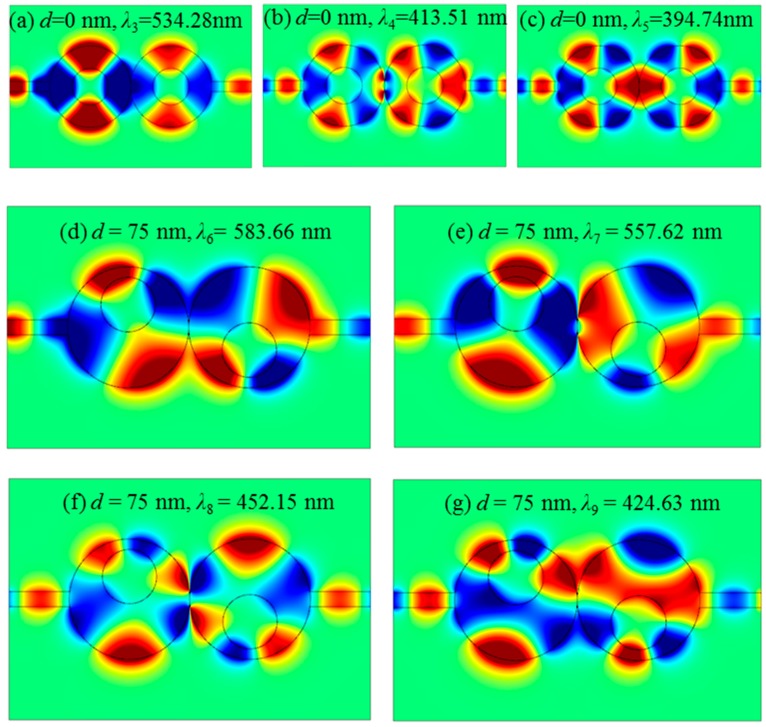
*H_z_* distributions of the symmetric structure with *d* = 0 at the resonance peaks of (**a**) *λ*_3_ = 534.28 nm; (**b**) *λ*_4_ = 413.51 nm; (**c**) *λ*_5_ = 394.74 nm; and the asymmetric structure with *d* = 75 nm at the resonance peaks of (**d**) *λ*_6_ = 583.66 nm; (**e**) *λ*_7_ = 557.62 nm; (**f**) *λ*_8_ = 452.15 nm; and (**g**) *λ*_9_ = 424.63 nm.

In order to obtain further physical insights into the Fano resonances, we studied the *H_z_* distributions of the designed structures. Figure 5a–c shows the *H_z_* distributions of the symmetric case (*d* = 0) at the resonance peaks of *λ*_3_ = 534.28 nm, *λ*_4_ = 413.51 nm and *λ*_5_ = 394.74 nm. For the symmetry-breaking structure, we chose the case of *d* = 75 nm for an example. Figure 5d–g shows the *H_z_* distributions at the resonance peaks of *λ*_6_ = 583.66 nm, *λ*_7_ = 557.62 nm, *λ*_8_ = 452.15 nm and *λ*_9_ = 424.63 nm, respectively. It indeed shows that some new coupling patterns arise between the two cascaded cavities with the introduction of an asymmetric structure.

**Figure 6:**
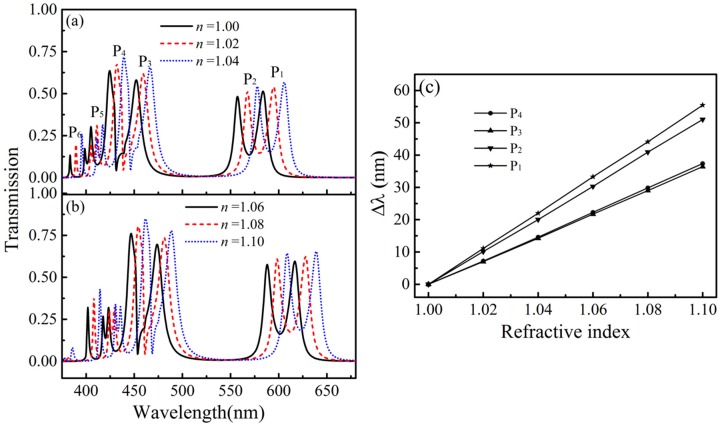
(**a**) Transmission changes for different refractive indexes *n* = 1.0, 1.02, 1.04; and (**b**) *n* = 1.06, 1.08, 1.10; (**c**) Corresponding wavelength changes of the resonance peaks for different refractive indexes.

**Figure 7:**
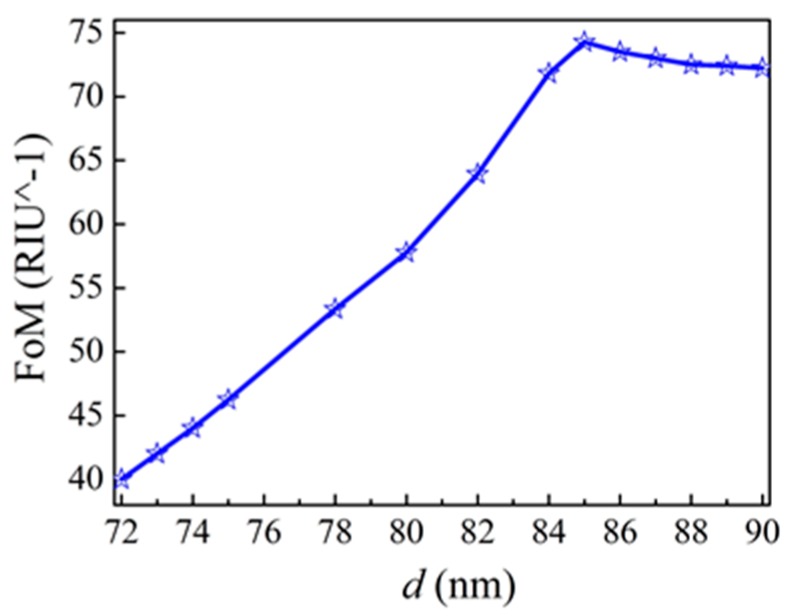
The changes of the figure of merit (FoM) value (resonance peak *P*_1_) related to the parameter of *d*.

Next, we will investigate the changes of transmission spectra for different refractive indexes *n* of the insulator in the structure, as shown in Figure 6a,b. We also chose the parameter *d* = 75 nm resonance peaks all redshift. As shown in Figure 6c, the resonance peaks of *P*_1_, *P*_2_, *P*_3_, and *P*_4_ redshift from 583.50 nm to 638.97 nm, from 557.48 nm to 608.51 nm, from 452.17 nm to 488.59 nm, and from 424.52 nm to 461.89 nm, respectively. Here, to evaluate the sensing performance of our structure, we also calculate the figure of merit (FoM), which is defined as the resonance wavelength shift upon the change of the refractive index of the surrounding medium *n*, divided by the resonance width at half-maximum: FoM = (δ*λ*/δ*n*)/Δ*λ*. The calculated FoM values for the four resonance peaks *P*_1_–*P*_4_ (see Figure 6c) are 46.23, 45.90, 22.14 and 36.35, respectively. Comparing the four resonance peaks of *P*_1_–*P*_4_, *P*_1_ is more sensitive to changes of refractive indexes than the other resonance peaks. The calculated results show that the resonance peak *P*_1_ has the maximum FoM of 46.23. For the resonance peak P_1_, we also calculate the FoM value related to the parameter of *d*, as shown in Figure 7. We can observe that, at first the FoM value becomes larger with the increase of parameter *d*. Then it reaches the maximum value as 74.3 at *d* = 85 nm. After the peak, the FoM value then decreases with the increase of parameter *d*. The reason for the reduction is mainly due to the transmission becomes bad for resonance peak *P*_1_. The FoM value of 74.3 is higher than the previous reported results [27,37].

## 4. Conclusions

In summary, we propose a new type of plasmonic sensor composed of a main waveguide and two cascaded asymmetric cavities at the center. The research results show that the cascaded asymmetric structure is advantageous for obtaining sharp Fano resonance peaks with high transmission through strengthening of the mutual coupling of the cavities. A maximum FoM value of 74.3 can be obtained by modulating the geometrical parameters appropriately. The plasmonic nanostructure presented here can be found in potential applications in biological/chemical sensors, optical filters, modulators, and other photonic integrated devices.

In addition, we note that the chirality in asymmetric structure is an interesting issue for many applications, such as designing switches to modulate optical circuits [38]. In our next work, we will pay more attention to this issue.
